# CD155 and Its Receptors as Targets for Cancer Therapy

**DOI:** 10.3390/ijms241612958

**Published:** 2023-08-19

**Authors:** Rossella Paolini, Rosa Molfetta

**Affiliations:** Department of Molecular Medicine, Laboratory Affiliated to Istituto Pasteur Italia-Fondazione Cenci Bolognetti, Sapienza University of Rome, 00161 Rome, Italy; rossella.paolini@uniroma1.it

**Keywords:** tumor immune surveillance, poliovirus receptor, cancer therapy

## Abstract

CD155, also known as the poliovirus receptor, is an adhesion molecule often overexpressed in tumors of different origins where it promotes cell migration and proliferation. In addition to this pro-tumorigenic function, CD155 plays an immunomodulatory role during tumor progression since it is a ligand for both the activating receptor DNAM-1 and the inhibitory receptor TIGIT, expressed on cytotoxic innate and adaptative lymphocytes. DNAM-1 is a well-recognized receptor involved in anti-tumor immune surveillance. However, in advanced tumor stages, TIGIT is up-regulated and acts as an immune checkpoint receptor, counterbalancing DNAM-1-mediated cancer cell clearance. Pre-clinical studies have proposed the direct targeting of CD155 on tumor cells as well as the enhancement of DNAM-1-mediated anti-tumor functions as promising therapeutic approaches. Moreover, immunotherapeutic use of anti-TIGIT blocking antibody alone or in combined therapy has already been included in clinical trials. The aim of this review is to summarize all these potential therapies, highlighting the still controversial role of CD155 during tumor progression.

## 1. Introduction

CD155/Necl5 is a type I transmembrane glycoprotein that belongs to the Nectins and Nectin-like (Necls) family of proteins, and it is also known as poliovirus receptor (PVR) since it allows poliovirus entry [1]. As with other members of this family, CD155 mediates important cellular processes, playing a critical role in adhesion, contact inhibition, migration, and proliferation [2,3].

Although CD155 is constitutively expressed at a low level in different tissues including vascular endothelial cells, spinal cord motor neurons, dendritic cells (DC), and macrophages [4,5,6], its overexpression correlates with poor prognosis in human malignancies of different origins such as non-small cell lung cancer (NSCLC), pancreatic cancer, sarcoma, melanoma, breast cancer, colorectal cancer, and multiple myeloma (MM) [7,8,9,10,11,12,13,14,15]. These observations suggest that CD155 expression may confer to tumor cell more aggressive features. When expressed on tumor cells, CD155 can trigger proliferative signals and favor tumor cell migration and metastasis [3,16,17].

CD155 also plays and immunoregulatory function. Indeed, it interacts with the activating receptor DNAX-associated molecule-1 (DNAM-1) expressed on the surface of cytotoxic T cells and natural killer (NK) cells, thus favoring tumor cell recognition and elimination by innate and adaptive immune responses [2,18]. On the other hand, CD155 also binds checkpoint inhibitory receptors, including T cell immunoreceptor with Ig and ITIM domains (TIGIT) and T cell-activated increased late expression (Tactile or CD96), thus counteracting DNAM-1 action in the late phases of tumor progression [19].

Although the clinical significance of CD155 expression in cancer remains controversial, this molecule represents a potential target of anti-tumor therapies. The scope of this review is to summarize approaches that direct target CD155 either as receptor for Poliovirus to address oncolytic virus action on cancerous cells or as a ligand for DNAM-1 and TIGIT immune receptors to favor a more efficient anti-cancer immune response.

## 2. The Complex Role of CD155 in Tumor Progression

**Figure 1:**
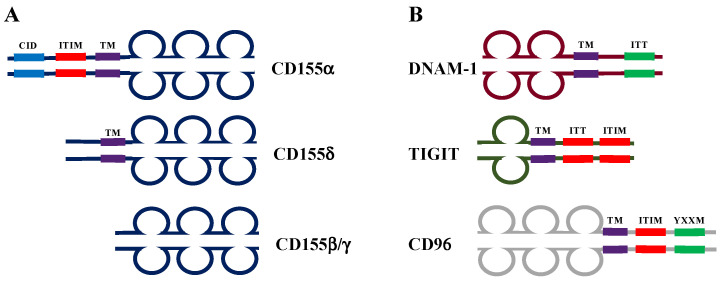
Schematic structure of human CD155 and its receptors. (**A**) Human CD155 is produced as four splice variants that differ in the presence of the transmembrane domain (TM) and the length of the C-terminal domain. This latter domain in hCD155α comprises an ITIM domain that initiates inhibitory signals and a domain interacting with clathrin adaptor complex (CID). (**B**) CD155 receptors include DNAM-1, TIGIT, and CD96 that harbor motifs of potential importance for signaling, as described in the text. Activating domains are depicted in green, whereas inhibitory domains are depicted in red.

CD155 is an adhesion molecule belonging to the IG superfamily of proteins with three Ig domains in its extracellular domain, as depicted in Figure 1A [3,20]. It is expressed at low levels in healthy tissue, but during malignant transformation, different signals and transcriptional programs are responsible for CD155 up-regulation [21,22,23]. This up-regulation may represent an advantage for cancer cells since CD155 contributes to tumor growth and the acquisition of an invasive phenotype [16,17]. These data, together with high CD155 expression in advanced clinical stage of human malignancies, mainly support a role for CD155 as a pro-tumorigenic molecule [7,8,9,10,11,12,13,14,15]. However, CD155 also exerts an anti-tumorigenic role regulating immune response to tumors thanks to its ability to interact with immune receptors on cytotoxic lymphocytes [18,19].

The results discussed in the following subsections and paragraphs outline the CD155 complex role in tumor initiation and progression, as well as its role in tumor immune surveillance.

### 2.1. Regulation of CD155 Expression and Function in the Tumor Microenvironment

Accumulating evidence demonstrates that CD155 expression may be regulated at transcriptional, post-transcriptional, and post-translational levels.

CD155 overexpression is regulated by signals implicated in tumor transformation, including the activation of the Ras-Raf-MEK-ERK pathway either by *Ras* mutations or fibroblast growth factor receptor stimulation [22]. Moreover, in MM, DNA damage has been shown to up-regulate CD155 expression through a pathway involving ataxia telangiectasia mutated (ATM) and Rad3-related (ATR) protein kinases [23,24,25].

Regarding the transcription factors involved, CD155 expression may be induced by Gli that is activated by the Sonic Hedgehog pathway [21], as well as NF-κB activated by different stimuli including Toll-like receptor ligands [26,27,28,29]. Recent studies demonstrate that cytokines may play a role in the modulation of CD155 expression during tumor transformation. Indeed, a role for T cell-derived IL-22 in the induction of murine CD155 expression on lung and breast cancer cells has been envisaged [30], while IL-8 derived from bone marrow stromal cells regulates CD155 expression in human MM [28].

Once transcribed, human CD155 mRNA can be alternatively spliced into different isoforms, resulting in the presence of four proteins sharing the same extracellular domains (Figure 1A) [31,32]: β and γ are soluble forms that lack the transmembrane domain and were found in different body fluids such as blood, cerebrospinal fluid, and urine; α and δ are transmembrane isoforms that contain different intracellular domains. In particular, the longest CD155α isoform possesses a unique intracellular domain that interacts with the clathrin adaptor complex and an immunoreceptor tyrosine-based inhibition motif (ITIM) responsible for signal transduction [33,34]. Whether different stressing stimuli regulate the expression of diverse CD155 isoforms in cancerous cells remains unknown.

Post-translational modifications including ubiquitin and ubiquitin-like pathways regulate CD155 expression in tumor cells [35,36]. Indeed, although the involvement of ubiquitin conjugation has not been formally demonstrated, CD155 is constitutively degraded in hepatocellular carcinoma cells by the activation of the unfolded protein response [37]. In MM cells, CD155 modification by the ubiquitin-like molecule SUMO promotes its intracellular retention resulting in a reduction of CD155 surface expression and an impairment of CD155-related functions including cytotoxic immune cell activation and MM adhesion to bone marrow stromal cells [17,38].

CD155 expression on cancerous cells has the potential to contribute to progression and spreading of the tumor thanks to the intrinsic ability of this molecule to induce proliferative signals and promote migration [39,40]. CD155-deficient mice showed reduced tumor burden in a model of colitis-induced colorectal cancer [41]. Accordingly, in human colorectal cancer cell lines, CD155 expression provides a proliferative advantage to the tumor while its knockdown shifts Bax/Bcl-2 balance towards pro-apoptotic signaling [42].

The molecular pathways underlying CD155-mediated proliferation are only partially clarified, mainly through experiments performed using NIH3T3 cells: CD155 synergizes with platelet-derived growth factor potentiating the Ras-Raf-MEK-ERK signaling pathway [43]. Of note, in cells in which Ras is constitutively active, CD155 overexpression is sufficient to promote cell proliferation. Conversely, CD155 knock-down results in cell cycle arrest [44]. Notably, the intracellular ITIM domain is required for CD155-induced proliferative signals [44], suggesting that this function is exclusively exerted by the CD155α isoform.

Even though findings in vivo are scarce, several pieces of in vitro evidence highlight a role for CD155 in regulating both cell–cell adhesion and cell migration. CD155 belongs to the Nectins and Nectin-like molecules (Necl), a family of Ig-like adhesion molecules able to bind to the extracellular matrix or to form homophilic and heterophilic interaction with members of the same family, contributing to the organization of adherent junctions [20]. Differently from Nectin proteins, CD155 does not form homophilic interactions but can bind to Nectin-3 [45,46] and vitronectin [47], thus mediating adhesion to neighboring cells and extracellular matrix, respectively. Upon interaction with Nectin-3, CD155 is rapidly internalized, allowing contact inhibition of cell movement and the formation of tight junctions [48]. This continuous internalization is counterbalanced by a strong CD155 up-regulation occurring during tumor progression. CD155 up-regulation allows novel but only partially defined interactions and favors cell migration. Of note, at the leading edge of migrating cells, CD155 colocalization with αvβ3 integrin induces Rac and Cdc42 signaling pathways that promote cytoskeleton reorganization and inhibition of focal adhesion [49].

Accordingly, CD155 expression enhances tumor spreading in primary gliomas, and its over-expression in glioma cell lines reduces adhesion to vitronectin and induces cell migration [50,51]. As demonstrated for proliferation, the CD155 ITIM domain appears to be required for the initiation of intracellular signals that inhibits adhesion and favors migration. Upon phosphorylation, this domain recruits the Src homology region 2 domain-containing phosphatase (SHP-2) that, in turn, dephosphorylates focal adhesion kinase (FAK) [34]. Although most of these results were not confirmed in vivo, CD155 expression in several cancers correlates with their metastatic potential [7,8,9,10,11,12,13,14,15].

### 2.2. CD155: A Double-Edged Sword in Cancer Immune Surveillance

CD155 up-regulation on the surface of cancerous cells provides an alert signal to both innate and adaptive immune cells. Indeed, CD155 is recognized by the activating receptor DNAM-1 or CD226 (Figure 1B), an immunoglobulin adhesion molecule expressed on most immune cells, including T cells, B cells, NK cells, and monocytes [18,52]. Mice lacking DNAM-1 expression are more prone to develop chemical-induced tumors and to the progression of transplanted tumors [53,54], highlighting the importance of this receptor in cancer immune surveillance. Interaction of DNAM-1 with its ligands, CD155 and CD112 (also known as Nectin-2), triggers activating signals able to co-stimulate NK and CD8^+^ T cell cytotoxic function [52,55,56]. Thus, CD155 expression renders tumor cells a more sensitive target of NK and CD8^+^ T cell elimination [57,58,59,60,61]. However, in advanced tumor stages, different mechanisms may contribute to dampening the DNAM-1/CD155 activation axis.

High amounts of soluble CD155 isoforms were found in biological fluids of patients with epithelial cancers and correlated with disease stages, representing a marker of poor prognosis [62]. It is likely that these isoforms compete with membrane-bound CD155 for DNAM-1 binding, decreasing DNAM-1-dependent cytotoxicity and facilitating tumor evasion from NK cell detection and elimination, as demonstrated in a murine melanoma model [63].

Moreover, CD155 expressed on tumor cells triggers DNAM-1 internalization and degradation in tumor infiltrating NK and T lymphocytes, promoting tumor metastatization [30,64]. Receptor down-modulation has also been observed on circulating NK cells of patients affected by different malignancies [60,65,66,67]. All together, these findings suggest that loss of the DNAM-1-mediated co-stimulatory signal is a key event leading to lymphocyte dysfunction in advanced tumor stages.

An additional mechanism that hampers immune surveillance in the tumor microenvironment is the expression on tumor-infiltrating cytotoxic cells of several checkpoint inhibitory receptors including cytotoxic T-lymphocyte antigen 4 (CTLA-4), programmed death receptor-1 (PD-1), T cell-immunoglobulin- and mucin-domain-containing molecule 3 (TIM-3), lymphocyte activation gene 3 (LAG-3), TIGIT, and CD96, which are all responsible for the acquisition of a dysfunctional phenotype [68,69]. To this regard, it is important to underline that recent evidence demonstrate that DNAM-1-mediated signaling may be dampened by the action of the checkpoint receptor PD-1. In particular, PD-1 is directly responsible for the activation of Src homology region 2 (SHP2) phosphatase that dephosphorylates the DNAM-1 intracellular domain, preventing signal propagation [70,71].

Notably, both TIGIT and CD96 are significantly up-regulated on NK and T cells that are chronically stimulated by tumor cells [72,73,74,75], and their expression is a hallmark of poor prognosis and resistance to chemotherapy [76]. On NK cells, the sustained engagement of activating receptors including NKG2D and NKp46 triggers TIGIT upregulation, decreasing NK cell functionality [75,77]. Both in humans and mice, TIGIT and CD96 compete with DNAM-1 for CD115 binding and bind to CD155 with higher affinity compared to DNAM-1 [78,79,80]. The molecular structure of CD155 receptors is schematized in Figure 1B. They all belong to the Ig superfamily since they contain a variable number of Ig domains in their extracellular sequence. DNAM-1 contains an ITT domain in its cytoplasmic tail that is responsible for the initiation of activating signals, whereas TIGIT and CD96 contain an ITIM domain, and, upon CD155 recognition, they trigger inhibitory signals that counterbalance DNAM-1-mediated activation [79,81,82]. In addition, human CD96 cytoplasmic tails also present the activation motif YXXM [83].

Besides the competitive binding, TIGIT may inhibit DNAM-1 with additional different mechanisms. It triggers inhibitory signals through its two cytoplasmic ITIM domains that, upon CD155 binding, recruit the inositol phosphatase SHIP-1, inhibiting PI3K-mediated signaling [78,84,85]. Moreover, TIGIT can directly bind to DNAM-1 in cis, limiting DNAM-1 ability to homodimerize and interfering with its co-stimulatory function in T cells [72]. TIGIT is also highly expressed on a subset of natural Tregs in both mice and humans and drives the acquisition of the immune suppressive phenotype [86]. These results suggest that TIGIT may suppress immune responses, promoting the differentiation of regulatory T cells.

TIGIT can also indirectly inhibit T cell functions, altering DC maturation. Indeed, upon TIGIT binding, CD155 promotes IL-10 secretion by DCs and prevents the production of the proinflammatory cytokine IL-12 [87].

Although CD96 was initially described as an adhesion molecule that facilitates the interaction between human NK cells and their targets [88], mice lacking CD96 display an increased NK cell functionality [82]. Moreover, the use of anti-CD96 blocking antibodies results in an increased NK cell-mediated immune surveillance in different murine models of metastatic cancers [89,90], highlighting an inhibitory role for this receptor on NK cells in vivo. Accordingly, more recent evidence in humans demonstrates that CD96 expression correlates with NK cell exhaustion and poor prognosis in hepatocellular carcinoma patients [74]. Regarding the role of CD96 in the regulation of T cell functionality, tumor growth is better controlled by CD96-negative CD8^+^ T cells compared to CD96-positive CD8^+^ T cells in murine models of colon cancer, melanoma, and fibrosarcoma [91]. However, CD96 can also function as a co-stimulatory molecule in CD8^+^ T cells since in vitro stimulation activates signaling pathways, leading to T cell proliferation both in humans and mice [92,93].

In conclusion, the role of CD96 in controlling NK and T cell activation is still debated. Of note, despite the presence of an ITIM domain, the cytoplasmic tail of human CD96 also contains a YXXM domain that confers to the molecule the potential to initiate activating signals by recruiting the p85 subunit of PI3 kinase [83]. Moreover, unlike murine CD96, the human receptor can be expressed in two splice variants that differ in the extracellular domain and bind to CD155 with different affinities [94]. However, future work is still necessary to understand whether the engagement of the two different human isoforms results in different functional outcome and whether in cancer patients, CD96 can be targeted to improve immune surveillance. Of note, a recent study in human tumor tissues revealed a correlation between CD96 expression and immune infiltration. However, depending on the histological tumor type, CD96 expression correlates with poorer prognosis or has a protective effect, suggesting that this molecule can exert a complex function depending on the tumor and the immune infiltration [95].

All together, these results highlight the importance of the CD155/TIGIT/CD96/DNAM-1 axis in cancer immune surveillance outcome.

## 3. Current Anti-Cancer Strategies Targeting CD155 and Its Receptors

**Figure 2:**
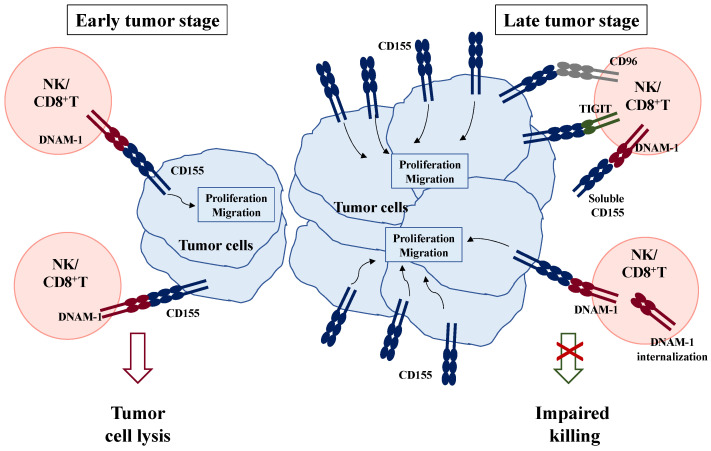
Model depicting CD155 roles during tumor progression. On the membrane of cancerous cells, CD155 engagement initiates intracellular signals, leading to proliferation and migration, favoring tumor growth and metastasis. However, in early phases of tumor transformation, CD155 also plays an anti-tumorigenic role, alerting the immune system against cancer. Indeed, it is recognized by DNAM-1 activating receptor expressed on NK and CD8^+^ T cells that mediates tumor cell killing (**left**). In late phases, DNAM-1 down-modulation from the surface of cytotoxic cells and a concomitant up-regulation of inhibitory CD155 receptors including TIGIT and CD96 contribute to dampen anti-tumor immune responses (**right**).

CD155 role in tumor progression is summarized in Figure 2. In the early tumor stage, CD155 represents a danger signal that alerts the immune system against cell transformation (Figure 2, left). However, CD155 up-regulation in transformed cells may represent an intrinsic factor that facilitates tumor growth and spreading. On cancer cells, it works as an adhesion molecule that modulates proliferation and increases cell migration. On the other hand, in later tumor stages, DNAM-1 internalization and the concomitant increased expression of inhibitory receptors renders immune cells unable to fight against cancer (Figure 2, right). In particular, TIGIT and CD96 preferentially bind to CD155 and transduce inhibitory signals that in turn suppresses DNAM-1-mediated cytotoxic function. Thus, the high levels of both CD155 and TIGIT may contribute to create an immunosuppressive environment, representing a mechanism of tumor evasion.

**Figure 3:**
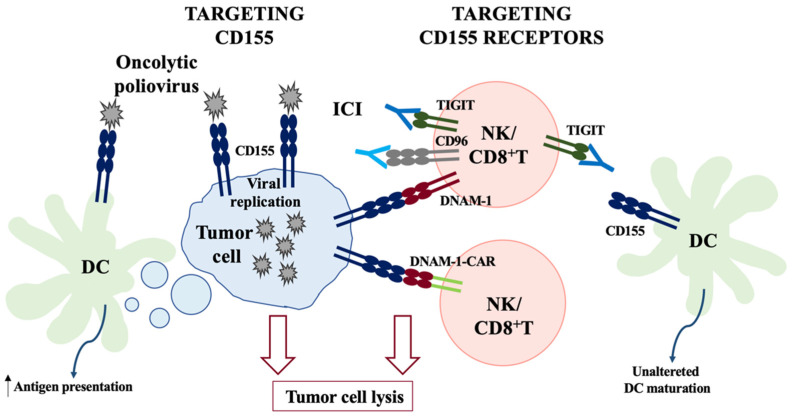
Therapeutic approaches targeting CD155 and its receptors. (**Left**): The direct targeting of CD155-expressing tumors can be achieved using engineered oncolytic polioviruses that result in tumor cell lysis. Dendritic cells (DCs) will capture apoptotic bodies, increasing their capability to present tumor antigens. DCs also express CD155, and they can be directly infected by oncolytic poliovirus. Infection induces type-I interferon production and increases tumor antigen presentation, promoting tumor-specific CD8+ T cell priming. (**Right**): Anti-tumor immune response may be boosted, potentiating DNAM-1 activation. To this aim, two therapeutic strategies have been exploited to increase tumor cytotoxicity by Natural Killer (NK) and CD8^+^ T lymphocytes (**right panel**). One is the use of blocking antibodies against the inhibitory receptors TIGIT and CD96 (ICI), allowing the interaction between CD155 and DNAM-1 (upper part of the right panel). This treatment will also prevent CD155 stimulation on DCs, allowing their canonical maturation. As an alternative approach, DNAM-1 can be fused to potent activating cytoplasmic domains to engineer a chimeric antigen receptor (DNAM-1/CAR) able to enhance effector functions of NK or CD8^+^ T cells in adoptive transfer therapies (lower part of the right panel).

All together, these finding strongly support the rationale for the development of therapeutic approaches targeting the CD155/DNAM-1/TIGIT axis, summarized in Figure 3 and discussed below.

### 3.1. Oncolytic Viruses Targeting CD155

Since CD155 represents the cellular receptor for poliovirus, a therapeutic approach is based on the use of engineered polioviruses (Figure 3, left). Indeed, they are oncolytic viruses with a natural tropism for CD155-expressing cells, and their selective replication in tumor cells is the first requirement for therapeutic efficacy [96,97]. Moreover, CD155 is expressed at low levels on healthy tissues while is overexpressed on tumor cells, thus minimizing side effects of oncolytic viruses.

Poliovirus oncolytic immunotherapy was initially developed as novel approach to treat pediatric brain tumors, including glioblastoma. The first oncolytic virus developed to exploit CD155 expression in glioblastoma was an engineered neuro-attenuated poliovirus called PVSRIPO [98]. Since poliovirus infects epithelial cells of the gastrointestinal tract and spinal cord motor neurons, causing poliomyelitis, this virus was attenuated, replacing the internal ribosome entry site (IRES) with the IRES from the human rhinovirus to reduce neurotoxicity [99]. However, the replication of this recombinant virus was observed also in the kidney, where poliovirus normally does not replicate. Thus, a new recombinant PVSRIPO virus was constructed with the aim to prevent replication in healthy cells, preserving the ability to lyse tumors, and its safety was confirmed in murine models [98]. Due to the high expression of CD155 in glioma cells, oncolytic poliovirus therapy entered phase I clinical trials. Intratumoral delivery of PVSRIPO in adult glioblastoma patients resulted in durable responses observed both by clinical signs and radiographic observation [100]. More recently, intratumoral injection of this recombinant poliovirus resulted safe in recurrent pediatric high-grade glioma in phase I of experimentation [101], and it has also been evaluated for treatment of pediatric neuroblastomas [102,103]. Moreover, preclinical animal models demonstrate the possible application of these viruses in other kinds of solid tumors, including breast and prostate cancers [104], as well as in human bone and soft tissue sarcomas [105]. A phase I clinical trial with intratumoral injection of PVSRIPO was also carried out in refractory melanoma patients, with a complete response in about 50% of patients [106].

Another important feature of oncolytic viruses is that they are designed to be immunogenic. Indeed, upon PVSRIPO infection, the lysis of tumor cells releases damage- and pattern-associated molecular patterns (DAMPs and PAMPs) recognized by innate immune cells such as neutrophils and DCs, which might act either directly by promoting transformed cell clearance [104] or indirectly through the activation of anti-tumor specific T cells [103]. Since DCs express CD155, they can also be infected by PVSRIPO. However, infection does not result in DC death but induces type I interferon production and increases tumor antigen presentation, promoting tumor-specific CD8^+^ T cell priming [107,108]. The role of DCs in oncolytic virotherapy is also depicted in Figure 3 (left). A role for tumor-associated macrophages (TAM) has also been recently envisaged in fresh tissue specimens derived from glioblastoma patients [109]. They are the main population in the tumor microenvironment infected by the PVSRIPO virus and play a crucial role in the production of cytokines initiating anti-tumor response.

In summary, preclinical studies demonstrate a potential success of oncolytic poliovirus-based anti-tumor therapy.

### 3.2. DNAM-1 Chimeric-Receptor-Based Therapies

One of the most promising therapies that revolutionized anticancer treatment is the adoptive transfer of patient-derived, ex vivo engineered T cells. These cells are designed to express a chimeric antigen receptor (CAR) that specifically recognizes tumor antigens. CARs usually contain an extracellular antibody-derived variable fragment (scFV) and an intracellular domain designed to drive activating signals, typically the intracellular domain of TCR/CD3 ζ-chain with the addition of co-stimulatory domains. This specific targeting boosts the ability of the T lymphocyte to kill tumor cells and has shown remarkable improvement of prognosis in several clinical trials, with the best results obtained in hematological malignancies [110]. Therefore, the development of new approaches improving this therapy in solid tumors is imperative.

Due to the high CD155 expression in different cancers, the use of DNAM-1 extracellular domain to target CD155-expressing tumors has been exploited (Figure 3, right). Since DNAM-1 is also involved in transendothelial migration [111], the expression of this receptor may enhance extravasation and trafficking in the tumor microenvironment of the engineered T cells. The DNAM-1 extracellular domain fused to the intracellular TCR ζ-chain-transducing domain was used to engineer T cells in a murine model of melanoma. This construct elicited high levels of cytotoxicity in vitro and reduced tumor growth in a murine melanoma model in vivo. However, for unknown reasons, cytokine production was low and not enhanced by the introduction of co-stimulatory motives [112].

Besides engineered T cells, the possibility to use NK cells for adoptive transfer therapy has been more recently exploited. The advantages of NK cell-based therapy are the lower costs and the possibility to use allogenic cells that, compared to autologous ones, have shown higher safety due to low risk of proliferation upon transfer. Moreover, since both NK and T cells derived from cancer patients are often dysfunctional, the use of allogenic cells allows for the overcoming of this limitation [113]. Preclinical studies have demonstrated the enhanced ability of NK cells to lyse neuroblastoma cell lines upon transfection with a CAR receptor based on the extracellular domain of DNAM-1 fused to the TCR ζ-chain-transducing domain [114]. This finding suggests that DNAM-1 may be employed in both NK and T cell-based adoptive transfer also for the treatment of solid tumors.

### 3.3. Monoclonal Antibodies Targeting CD155 Inhibitory Receptors

One of the typical hallmarks of the tumor microenvironment is the up-regulation of inhibitory receptors including CTLA-4, TIM-3, PD1, LAG-3, and TIGIT on the surface of cytotoxic lymphocytes. Upon T cell activation, most of these receptors are physiologically up-regulated and play a key role in the maintaining of self-tolerance. However, in the tumor microenvironment, their overexpression renders immune cell functionally unable to fight cancer [68,69]. Thus, the employment of monoclonal antibodies (mAbs) able to block the activity of the checkpoint inhibitory receptors, namely immune checkpoint inhibitors (ICI), offers great potential for tumor control. Several ICI, including anti-CTLA-4 [115] and anti-PD-1 [116] antibodies, have already been approved and have shown promising results. Moreover, the possibility to use therapeutic antibodies raised against the ligands of these checkpoint receptors has also been exploited with antibodies targeting PD-L1, the ligand for PD-1 expressed on tumor cells [117,118]. However, since some patients display resistance to these therapies, the identification of new therapeutic targets is critical [68]. Therefore, the TIGIT/CD155 axis raised great interest as a novel target for ICI treatments (Figure 3, right) [81]. A first paper demonstrating enhanced killing of tumor cells upon TIGIT inhibition employed a polyclonal anti-TIGIT antibody in vitro [79]. More recently, several human ex vivo studies and the use of in vivo murine models have demonstrated the efficacy of anti-TIGIT treatment. In particular, the reversion of T cell exhaustion accompanied by a subsequent improvement in immune surveillance and tumor rejection was observed upon treatment with anti-TIGIT mAbs in combination with other ICI [72,119,120]. For instance, the simultaneous inhibition of TIGIT and PD-L1 synergized to enhance tumor-infiltrating CD8^+^ T lymphocyte functions and promoted the rejection of transplanted tumors both in a colon cancer xenograft mouse model and in a syngeneic murine model of breast carcinoma [72]. TIGIT is also co-expressed with PD-1 in circulating CD8^+^ T cells of metastatic melanoma cells, and their concomitant blockade enhances tumor-specific T cell proliferation, cytokine production, and degranulation [119]. More recently, the use of the anti-TIGIT blocking antibody tiragolumab, together with PD-1, blocking in colorectal cancer, turned out to be an efficient therapy able to restore the functionality of tumor-infiltrating CD8^+^ T cells [120].

Regarding NK cells, the selective TIGIT blockade was sufficient to impair tumor growth [73], demonstrating a pivotal role for NK cell-mediated tumor rejection upon anti-TIGIT therapies. Several mAbs targeting TIGIT interaction with CD155 are currently in clinical trials and have displayed promising results in NSCLC, melanoma, and other solid tumors (Table 1).

One of the first compounds that has been entered clinical trials is tiragolumab. It is a fully human IgG1/kappa monoclonal antibody that blocks TIGIT/CD155 binding, and it was used in combination with atezolizumab (anti-PD-L1 mAb) in recurrent or metastatic NSCLC. Of note, the combined use revealed a better response compared to PD-1/PD-1L inhibition alone. This trial completed the phase II of experimentation, while phase III is currently ongoing. An improvement of progression-free survival compared with placebo plus atezolizumab has already been shown [121]. Vibostolimab is a humanized IgG1 mAb, and it represents another anti-TIGIT compound currently in phase II of clinical trials in patients with advanced NSCLC and melanoma in combination with anti-PD-1 pembrolizumab [122], while domvanalimab is another humanized IgG1 anti-TIGIT antibody that in preclinical studies improved T cell functionality when used alone or in combinations with PD-1 blockade. It is currently in phase II of clinical trial in NSCLC and phase III for metastatic NSCLC [123].

Other anti-TIGIT blocking antibodies include etigilimab, which was employed in combination with the anti-PD-1 nivolumab in metastatic solid tumors and is currently in phase II of clinical trials [124]; the humanized IgG1 ociperlimab, which is in phase II or III in different solid tumors in combination with PD-1 blocking; and EOS-448, BMS-986207, ASP8374, COM902, and IBI939, which entered phase I of clinical trials more recently [123].

Moreover, in a preclinical murine model of lung cancer, TIGIT blockade can increase the efficacy of adoptive transfer therapies with engineered T cells [125], suggesting that, besides anti-PD-1 or PD-L1, the specific TIGIT targeting may be combined to other anti-cancer therapies.

Regarding CD96, even though its role in humans remains controversial, preclinical studies demonstrate that the use of mAbs blocking CD96 interaction with CD155 may be exploited in anti-cancer therapy. Indeed, an increase in the efficacy of anti-PD-1 in combination therapy with anti-CD96 blocking mAbs have been observed in murine and human cancers [89,90,126,127]. However, future studies are needed to assess the potential role of CD96 as a target for cancer immunotherapy. Therefore, CD96-associated clinical trials in cancer patients are still lacking.

## 4. Concluding Remarks and Future Perspectives

CD155 has raised increasing interest in last years, and it is now considered a target for cancer therapies, comparable to PD-L1.

Although approaches that block CD155 interaction with the inhibitory receptor TIGIT have already obtained remarkable results in clinical trials, an open question is whether a direct target of CD155 can efficiently reduce tumor burden in vivo. For instance, the development of anti-CD155 antibodies that inhibit the intrinsic pro-tumoral function of CD155, but not its interaction with DNAM-1, may limit tumor growth and metastasis. Promising results have been obtained using an anti-CD155 antibody to limit tumor metastasis in osteosarcoma by blocking CD155-induced signal transduction [11].

Moreover, a deeper understanding of how CD155 expression and function are regulated in tumor cells may open the possibility to target selective player(s)/pathway(s) involved in tumor cell proliferative and migrating ability. To this regard, a recent study demonstrated that FAK kinase inhibition blocks CD155-induced signaling [128]. It would also be interesting to learn more about the posttranslational regulation of CD155. For example, understanding whether CD155 phosphorylation and/or SUMOylation differentially affect CD155 α and δ isoforms in terms of expression and function may shed light on new targets for tumor control.

An additional issue would be to elucidate how CD155 regulates immune responses by interacting with DNAM-1, CD96, and TIGIT. An open question is whether CD155 interaction with DNAM-1 is more important in early tumor development rather than in more advanced and metastatic stages. One possibility could be to target pathways that regulate CD155 expression only at later stages.

Finally, a still unexplored therapeutic strategy may be a combined use of oncolytic polio virotherapy and checkpoint inhibition to evaluate whether they may have a synergistic effect.

In conclusion, future studies are needed to explore new therapeutic strategies aimed to boost immune response against CD155 positive cancers.

## Figures and Tables

**Table 1:** List of anti-TIGIT antibodies currently in clinical trials, according to the official website www.clinicaltrial.gov (accessed on 9 August 2023).

Therapeutic Agent	Tumor	Clinical Phase	Identification Number
Tiragolumab	NSCLC	III	NCT04294810
Vibostolimab	NSCLC Melanoma	I/II I/II	NCT04165070 NCT04305041
Domvanalimab	NSCLC Metastatic NSLC	II III	NCT04262856 NCT04736173
Etigilimab	Metastatic solid tumors	I/II	NCT04761198
Ociperlimab	Cervical cancer NSCLC Esophageal Squamous Cell Carcinoma	II III II	NCT04693234 NCT04746924 NCT04732494
EOS-448	Advanced cancers	I	NCT04335253
BMS-986207	Multiple Myeloma Solid tumors	I/II	NCT04150965 NCT02913313
ASP8374	Solid tumors	I	NCT03260322
COM902	Advanced cancers	I	NCT04354246
IBI939	NSCLC	I	NCT04672369
